# Reply to the letter from Professor Hardell

**Published:** 1993-05

**Authors:** J.G. Smith, A.J. Christophers


 © Macmillan Press Ltd., 1993

LETTER TO THE EDITOR

### Reply to the letter from Professor Hardell

**Sir** – Professor Hardell has pointed out several methodological problems in our case control study, all of which were discussed in detail in our paper (Smith & Christophers, 1992). One of his criticisms is partly unjustified in that he states that 'the interviewers were not blinded.' In fact, the interviewer was not blind as to the status of the population controls but was blind with respect to the case/control status of the patients with cancer. The 'administrative problems' on which Professor Hardell sought clarification were the problems which must occur in many epidemiological studies, namely changing staff and delays while working on other projects. They were the main cause of the lengthy duration of the study.

Professor Hardell referred to our relative risks of 2.0 (soft tissue sarcoma) and 2.7 (malignant lymphoma) for those exposed to phenoxy herbicides or chlorophenols for more than 30 days. However these relative risks were not statistically significant; the lower 95% confidence limits were 0.5 and 0.7 respectively. The study was designed to detect increased risks from exposures of at least 1 day to phenoxy herbicides or chlorophenols. In order to adequately test hypotheses regarding exposure of more than 30 days, a much larger study would be required because very few people are exposed to this extent.

Professor Hardell's review of the literature is somewhat selective and he has ignored the vast majority of studies which have failed to find an association between exposure to phenoxy herbicides and malignant lymphoma. The authors of the two major combined cohort studies on 2,3,7,8-tetrachlorodibenzo-*p*-dioxin were more cautious in their conclusions concerning soft tissue sarcoma than Professor Hardell has claimed (Fingerhut *et al.*, 1991; Saracci *et al.*, 1991).

We agree with Professor Hardell's concluding paragraph that studies which are based on actual exposure are more useful than studies based on surrogates for exposure such as job category. Our study was based on detailed occupational and exposure histories obtained in face-to-face interviews with the subjects themselves. By using this method we think we have obtained accurate exposure data.

Yours etc,

J.G. Smith,
Statistical Centre,
Peter MacCallum Cancer Institute,
481 Little Lonsdale Street,
Melbourne, Victoria, Australia 3000.
A.J. Christophers,
Department of Pharmacology,
University of Melbourne,
Victoria, Australia 3052.

**References**

FINGERHUT, M.A., HALPERIN, W.E., MARLOW, D.A., PIACITELLI, L.A., HONCHAR, P.A., SWEENEY, M.H., GREIFE, A.L., DILL, P.A., STEENLAND, K. & SURUDA, A.J. (1991). Cancer mortality in workers exposed to 2,3,7,8-tetrachlorodibenzo-*p*-dioxin. *New Engl. J. Med.*, **324**, 212–218.

SARACCI, R., KOGEVINAS, M., BERTAZZI, P., BUENO DE MESQUITA, B.H., COGGON, D., GREEN, L.M., KAUPPINEN, T., L'ABBÉ, K.A., LITTORIN, M., LYNGE, E., MATHEWS, J.D., NEUBERGER, M., OSMAN, J., PEARCE, N. & WINKELMANN, R. (1991). Cancer mortality in workers exposed to chlorophenoxy herbicides and chlorophenols. *Lancet*, **338**, 1027–1032.

SMITH, J.G. & CHRISTOPHERS, A.J. (1992). Phenoxy herbicides and chlorophenols: a case control study on soft tissue sarcoma and malignant lymphoma. *Br. J. Cancer*, **65**, 442.


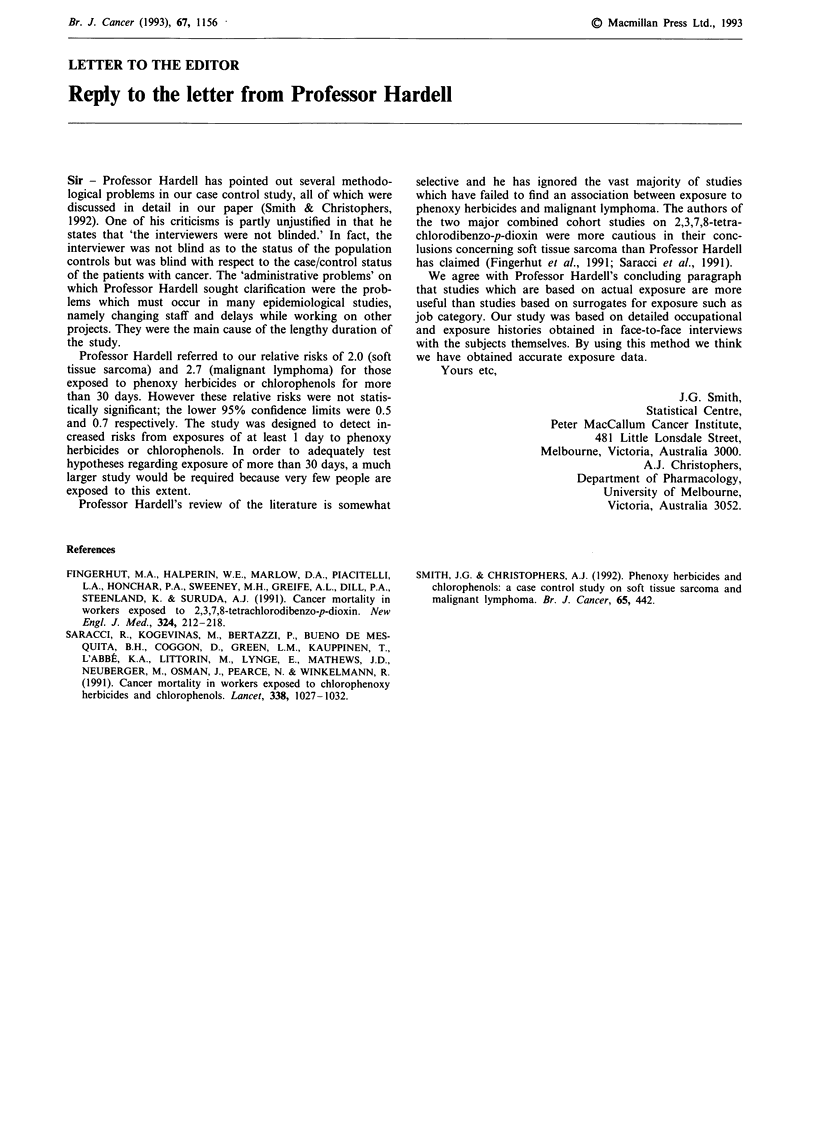


## References

[OCR_00068] Fingerhut M. A., Halperin W. E., Marlow D. A., Piacitelli L. A., Honchar P. A., Sweeney M. H., Greife A. L., Dill P. A., Steenland K., Suruda A. J. (1991). Cancer mortality in workers exposed to 2,3,7,8-tetrachlorodibenzo-p-dioxin.. N Engl J Med.

[OCR_00077] Saracci R., Kogevinas M., Bertazzi P. A., Bueno de Mesquita B. H., Coggon D., Green L. M., Kauppinen T., L'Abbé K. A., Littorin M., Lynge E. (1991). Cancer mortality in workers exposed to chlorophenoxy herbicides and chlorophenols.. Lancet.

[OCR_00083] Smith J. G., Christophers A. J. (1992). Phenoxy herbicides and chlorophenols: a case control study on soft tissue sarcoma and malignant lymphoma.. Br J Cancer.

